# Multilocus sequence analysis reveals high genetic diversity in clinical isolates of *Burkholderia cepacia* complex from India

**DOI:** 10.1038/srep35769

**Published:** 2016-10-21

**Authors:** Vikas Gautam, Prashant P. Patil, Sunil Kumar, Samriti Midha, Mandeep Kaur, Satinder Kaur, Meenu Singh, Swapna Mali, Jayanthi Shastri, Anita Arora, Pallab Ray, Prabhu B. Patil

**Affiliations:** 1 Department of Medical Microbiology Post Graduate Institute of Medical Education and Research, Chandigarh, India; 2 Bacterial Genomics and Evolution Laboratory, CSIR-Institute of Microbial Technology, Chandigarh, India; 3 Department of Paediatrics, Post Graduate Institute of Medical Education and Research, Chandigarh, India; 4 Department of Microbiology, Topiwala National Medical College & B. Y. L. Nair Charitable Hospital, Mumbai, India; 5 Fortis Escorts Heart Institute, New Delhi, India

## Abstract

*Burkholderia cepacia* complex (Bcc) is a complex group of bacteria causing opportunistic infections in immunocompromised and cystic fibrosis (CF) patients. Herein, we report multilocus sequence typing and analysis of the 57 clinical isolates of Bcc collected over the period of seven years (2005–2012) from several hospitals across India. A total of 21 sequence types (ST) including two STs from cystic fibrosis patient’s isolates and twelve novel STs were identified in the population reflecting the extent of genetic diversity. Multilocus sequence analysis revealed two lineages in population, a major lineage belonging to *B. cenocepacia* and a minor lineage belonging to *B. cepacia*. Split-decomposition analysis suggests absence of interspecies recombination and intraspecies recombination contributed in generating genotypic diversity amongst isolates. Further linkage disequilibrium analysis indicates that recombination takes place at a low frequency, which is not sufficient to break down the clonal relationship. This knowledge of the genetic structure of Bcc population from a rapidly developing country will be invaluable in the epidemiology, surveillance and understanding global diversity of this group of a pathogen.

*Burkholderia cepacia* complex (Bcc) is a complex group of non-fermenting Gram-negative bacilli (NFGNB) that comprises 20 validated species^1^. Bcc is the fourth most common pathogenic NFGNB worldwide after *Acinetobacter baumannii, Pseudomonas aeruginosa* and *Stenotrophomonas maltophilia*^2^. Bcc is widespread in natural and manmade habitats as these bacteria have a vast metabolic capacity^3^,^4^. Bcc is recognized as important opportunistic pathogens in immunocompromised, cystic fibrosis (CF), chronic granulomatous disease patients, and are also important nosocomial pathogens in hospitalized patients, causing life-threatening bacteraemia, urinary tract infections and respiratory tract infections because of high intrinsic antibiotic and disinfectant resistance^5^,^6^.

Various molecular typing methods are currently being employed for the identification and typing of Bcc, including whole cell protein analysis, amplified fragment length fingerprinting, whole cell fatty acid analysis, restriction fragment length analysis (RFLP), multilocus sequence typing (MLST)^7^,^8^. Comparatively a newer technique in clinical microbiology for identification of Bcc, matrix-assisted laser desorption ionization time-of-flight (MALDI-TOF) mass spectrometry is also being used nowadays^9^,^10^. Whereas all of these methods are able to discriminate Bcc from other NFGNB but not able to differentiate at species level. MLST is globally accepted method and provides high resolution at species level than other methods^8^. MLST scheme for Bcc was developed in 2005 and improved in 2009, which is globally used for typing and assessing the population structure of Bcc^11^,^12^. Multilocus sequence analysis (MLSA), which utilize phylogenetic procedure on the nucleotide sequences of allelic locus used in the MLST are being widely used for identification and phylogenetic relationships between isolates.

MLST is a powerful tool for typing and has provided insights into the population structure and recombination events in bacteria^13^,^14^. However, MLST studies on Bcc from developing countries in general and from India, in particular are lacking. With high patient diversity and density along with heavy use of antimicrobial agents, such studies are need of the hour. The present study involved MLST analysis of 57 clinical isolates of Bcc obtained during the time span of seven years (2005–2012) from four hospitals across India. For the first time in India, Bcc has been isolated from CF patients and three isolates from CF were included in this study^15^. Further identified the species of Bcc using MLSA, inferred about the genetic diversity and recombination features. This analysis has revealed novel insights of genetic diversity of Bcc isolates from India, which is important in epidemiological surveillance.

## Results

Total 57 Bcc isolates were included in this study. Out of these 57 isolates, 42 isolates were obtained from tertiary referral hospital of Post Graduate Institute of Medical Education and Research, Chandigarh, India (PGIMER; n = 42); 6 isolates from blood cultures from Fortis Escorts Heart Institute, New Delhi (FEHI; n = 6); 6 isolates from urine and blood culture were obtained from Topiwala National Medical College & B. Y. L. Nair Charitable Hospital, Mumbai (TNMC; n = 6) and 3 from blood cultures from Global Hospitals & Health City, Chennai (GHH; n = 3). A majority of isolates were obtained from blood cultures (n = 46) and rest from respiratory specimens (n = 6), endotracheal aspirate (ETA) (n = 2) and pus (n = 1), urine (n = 1) and body fluid (n = 1) from patient population having septicaemia, meningitis, peritonitis, pus, respiratory tract and urinary tract infections (Supplementary Table 1). Besides non-CF patients, three isolates are from CF patients, two of them were obtained from ETA and one from blood culture.

### E-MLST and population structure

**Figure 1:**
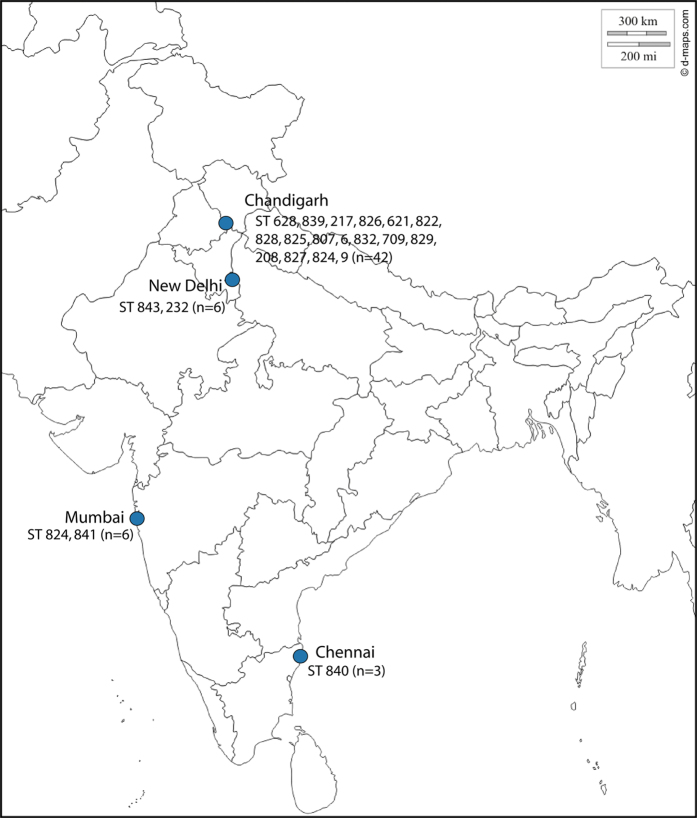
Geographical distributions of sequence types. A map of India showing locations of the hospitals from where the Bcc isolates were obtained, isolate number (n) and their sequence types identified. Template of India map has been adopted from the http://www.d-maps.com/ and URL for the respective map is http://d-maps.com/carte.php?num_car=4183&lang=en.

A total of 21 sequence types (ST), including 12 novel STs, which, harboured 11 novel alleles were identified amongst 57 Bcc isolates included in this study. Locations of the hospitals from where the Bcc isolates were obtained and their STs identified are shown in Fig. 1. The isolation details, ST identified along with the allelic profile of 57 Bcc isolates are mentioned in Supplementary Table 1.

**Figure 2:**
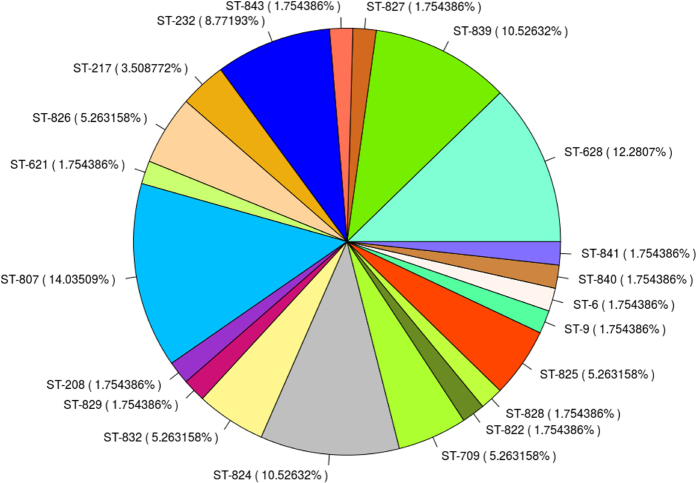
Abundance and sequence type diversity. Pie chart representing sequence type diversity and their abundance among the 57 Bcc isolates under study.

The epidemiological details of the prevalent STs based on E-MLST of non-CF isolates (n = 54) and CF isolates (n = 3) revealed 21 STs including 12 unique STs (822, 824, 825, 826, 827, 828, 829, 832, 839, 840, 841, & 843) and 11 novel alleles (*atp*D 339-40; *glt*B 393-4; gyrB 580-2, 585, 587-8, 593) were detected. STs 807 (14.03%), 628 (12.28%) and 839 (10.5 2%) were the most prevalent STs in isolates of this study, among them ST 839 was novel. The details on the abundance and distribution of the STs are mentioned in Fig. 2. Isolates 8947 and 9500 were isolated from the same patient at an interval of one week but had different sequence types as ST6 and ST628 respectively. Interestingly, in one case four serial isolates (4613, 5310, 5312 and 7216) were retrieved out from the same patient admitted in bone marrow transplant unit of PGIMER with two distinct novel STs (826 and 825). The isolates 4613, 5310 and 5312 with ST825 are the initial isolates and three weeks later isolate 7216 with ST-826 was isolated. Two novel STs (828, 826) were obtained from three CF isolates (1236, 7055 & 7716) under this study. Interestingly, ST 826 was also isolated from the blood culture of non-CF patient. Isolates 7055 and 7716 isolates were obtained from the same patient with a similar allelic profile (ST 826) but had different isolation sources, i.e. endotracheal aspirate and blood.

**Figure 3:**
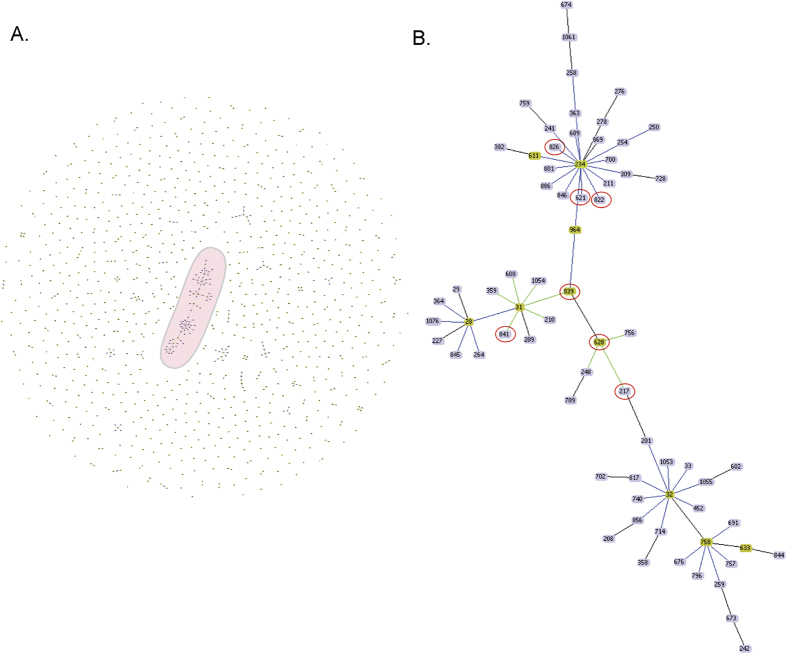
Populations structure analysis. (**A**) goeBURST analysis of the 1083 STs present in the PubMLST database. Each dot represents the single ST. Groups are formed by linking the STs that are double locus variants (DLV) and called as clonal complex (CC). The largest clonal complex 31 is highlighted in the pink eclipse. Light green - Group founder; Dark Green-Sub-group founder; Light blue - Common node. (**B**) Snapshot of the clonal complex 31 which is highlighted as a pink circle (in A). The STs which are marked as red colour circles are from this study which is linked to clonal complex 31.

21 STs found in this study were grouped into 5 BURST groups (BG) and 12 singleton STs by eBURST (Supplementary Table 2). The largest BG had 15 isolates and 3 STs (628, 839 and 217) with predicted founder ST 628, second largest group comprised of 6 isolates and 2 STs (232 & 843). Another group, which comprised of 5 isolates and 3 STs (826, 821 and 822) with predictor founder 826 and 13 unrelated STs, which were predicted to be singletons. goeBURST analysis with all 1083 STs present in the PubMLST database (as on 4^th^ September 2016) including STs under this study (Fig. 3A) revealed that the seven STs-628, 839, 217, 826, 621, 822 & 841 are linked to the 31 clonal complex (CC), which is the largest clonal complex (Fig. 3B). Among the STs that are linked to the 31CC, ST 826 was obtained from an isolate of CF patient.

### Sequence diversity

In order to assess the sequence diversity of the seven MLST loci of the isolates under study, average GC content was calculated, the number of polymorphic sites, nucleotide diversity (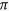), and ratio of non-synonymous (d_N_) to synonymous (d_S_) substitutions were determined for each MLST locus and mentioned in Table 1. The ratio of non-synonymous and synonymous substitutions (*d*_*N*_*/d*_*s*_) measures the level of selection in a protein-coding gene. The ratio of *d*_*N*_*/d*_*S*_ indicates purifying selection if *d*_*N*_*/d*_*S*_ <1, positive selection if *d*_*N*_*/d*_*S*_ >1, and neutral evolution if values are close to 1. The *d*_*N*_*/d*_*s*_ values ranging from the 0.000 (*trpB*) to 0.241249 (*atpD*) indicating that the all seven MLST loci exhibiting purifying selection. The mean GC content of the MLST gene fragments ranging from the 61.1% for *phaC* to 69.3% for the *trpB*. The nucleotide diversity index (π) ranging from the 0.00522 (*gltB*) to 0.02003 (*gyrB*). The number of polymorphic sites varied from the 13 for *gltB* to 46 for the *gyrB*, the most polymorphic locus.

### Multi locus sequence analysis

**Figure 4:**
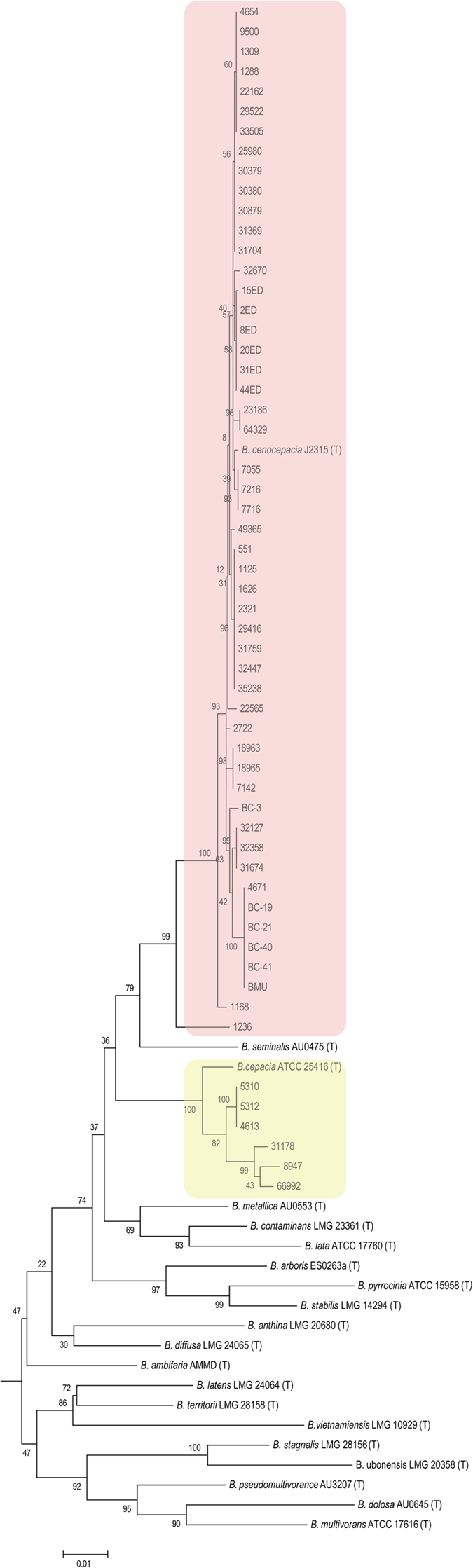
Phylogenetic analysis. Phylogenetic tree obtained from concatenated sequences of the MLST allelic loci with maximum likelihood method using MEGA 6.1. *B. cenocepacia* and *B. cepacia* lineages are highlighted with light red and yellow colour respectively.

All isolates included in this study are previously identified as Bcc by *recA*-RFLP. However, a major disadvantage of this method is that most species include multiple restriction profiles and overlapping of restriction profiles in multiple species which limits this method for accurate species identification of Bcc. Thus for the identification of species of the Bcc isolates we employed MLSA, which involves phylogenetic analysis on the nucleotide sequences of the alleles utilized in MLST. Phylogenetic analysis was carried out using multilocus sequence information of isolates under study by including the sequences from type or reference strain of 20 validated species of Bcc (Supplementary Table 3). The addition of reference strains or type strains in MLSA enables us the more accurate identification of the species of Bcc. The maximum likelihood tree based on seven loci showed that Bcc isolates under this study are grouped into two groups. First is a major group consisting of 51 isolates (89.47%), whose all 16 STs (including 8 novel) form one tight cluster along with *B. cenocepacia* J2315^T^ and belong to one lineage. The second is a minor group consisting of 6 isolates (10.53%) belonging to 4 novel STs (823, 838, 825 & 837) that cluster with *B. cepacia* ATCC 25416^T^. The resulting phylogenetic tree revealed that the major lineage belonged to *B. cenocepacia* while other minor but diverse lineage belonged to *B. cepacia* (Fig. 4). This suggests that the population of Bcc consist of only two species *B. cenocepacia* and *B. cepacia*. Interestingly, out of four serial isolates from the single patient, initial isolates (5310, 5312 and 4613) belong to *B. cepacia* lineage while the later isolate (7216) groups with the *B. cenocepacia* lineage. Similarly, amongst the isolates 8947 and 9500 from the same patient but initial isolate belongs to *B. cepacia* and later one to *B. cenocepacia* clad respectively. Three CF isolates including two from the same patient belong to the major *B. cenocepacia* lineage.

### Recombination analysis

**Figure 5:**
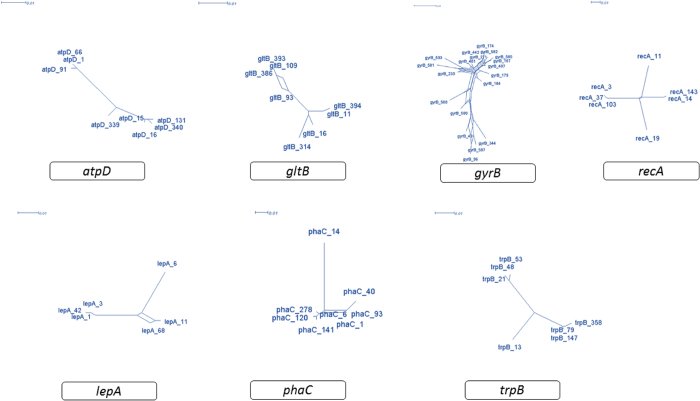
Split network analysis of seven individual MLST loci. Split network analysis of seven individual MLST loci. Multi-parallelogram formations indicate recombination events. The numbering in the figure refers to allele numbers.

**Figure 6:**
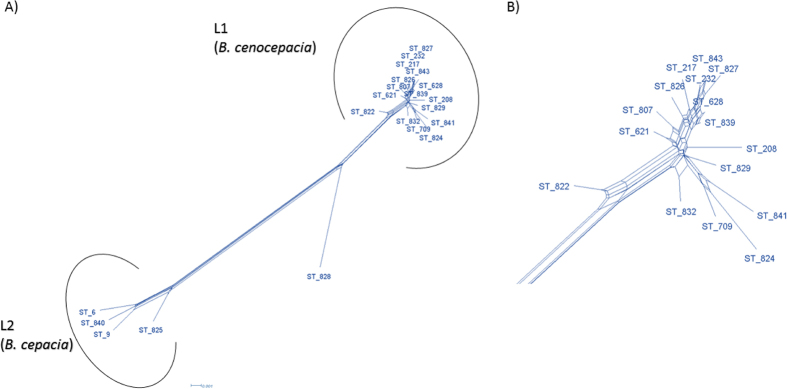
Split network analysis of concatenated sequences of MLST allelic locus of 21STs. Split network analysis based on the concatenated sequences of MLST allelic locus of 21STs identified among 57 Bcc isolates by neighbor-net method. The numbering in the figure refers to ST **(A)** Split network analysis of concatenated sequences of MLST allelic locus of 21 STs. **(B)** Split network analysis of *B. cenocepacia* lineage L1 showing multi parallelogram formation indicating evidence of recombination.

Split network analysis to examine the evidence for recombination amongst the 57 isolates revealed different structures in the split graphs for seven loci (Fig. 5). The split graphs for *gltB, gyrB, recA, phaC* and *lepA* revealed a network like with parallelogram structures indicating that intergenic recombination had occurred during the evolutionary history of these genes. However, the split graphs of *atpD* and *trpB* are tree-like structures suggesting that the descent of these genes was clonal and absence of recombination. The split decomposition analysis of combined seven MLST loci display network like structure with rays of different length (Fig. 6A). The 21 STs representing all isolates are divided into two main groups, L1 and L2 which corresponds to isolates of *B. cenocepacia* and *B. cepacia* respectively identified by the MLSA. Group L1 and L2 are completely disconnected from each other suggests that the interspecies recombination had not occurred. However, intergenic recombination has occurred between the isolates of *B. cenocepacia* group (L1) during the course of evolution as parallelogram-shaped groupings were detected (Fig. 6B). ST 828, which contains only one isolate 1236 from CF patient was clearly disconnected from both the groups suggesting that there is no recombination between this isolates and isolates of the other two group.

The *phi* test is a rapid statistically efficient test for recombination. The P value generated from *phi* test for all 21 STs is 5.923E-04 (Table 2) indicating significant incidence of recombination across the whole population. However, P value for the individual group *B. cenocepacia* (L1) and *B. cepacia* (L2) are 0.2992, 0.1748 respectively suggesting no significant evidence of recombination. The per site recombination/ mutation ratios (ρ/θ) values for all population and individual lineages are below 1 (Table 2) suggesting that the mutation occurred more often than recombination. Standard association of index (*I*_*A*_^*S*^) measures the homologous recombination by estimating the linkage disequilibrium between seven loci. The *I*_*A*_^*S*^ is expected to be zero when a population is at linkage equilibrium. Analysis of 21 STs under study yielded I_A_^S^ of 0.3828 (*P* = 1.00E-04) and 0.3828 (*P* = 1.00E-04), 0.2733 (P = 9.8E-02) for individual lineages *B. cenocepacia* (L1) and *B. cepacia* (L2) respectively. *I*_*A*_^*S*^ values are significantly different from zero suggesting linkage disequilibrium among the alleles indicating the clonal relationship and recombination was not sufficient to break down the linkage disequilibrium.

## Discussion

Multilocus sequence typing is a method of choice for the typing of Bcc due to its ability to differentiate the species in Bcc complex^5^,^8^,^16^,^17^. The MLST is also employed to study the global epidemiology of the Bcc isolates of CF^18^. Recently, E-MLST typing had been described that addressed the shortcomings in the original MLST methodology^12^, which was performed in this study on 57 clinical isolates of Bcc from India which was never studied before.

MLST successfully revealed the extent of diversity in Bcc isolates from a rapidly developing country like India where co-incidentally the patient density and diversity is also high, which is further coupled with the intense use of antibacterial agents^19^,^20^. In this study, 57 clinical isolates of Bcc are distributed in to 21 STs including 12 novel STs indicating the extent of diversity. Not only could we identify a large number of novel STs but half of the population belonged to these novel STs. In fact, one novel ST (ST824) constituted 10.6% of the population. The study has also provided first insights into the diversity of CF isolates from India where CF is not prevalent^21^,^22^. The fact that one of this novel ST from CF patient was also isolated from non-CF patient suggests the need of more exhaustive and exclusive MLST studies on CF isolates.

Among the Bcc, *B. cenocepacia* and *B. multivorans* together account 85–97% of all Bcc infections. Despite this, the other species of which rarely found in clinical infections are *B.cepacia, B. stabilis, B. vietnamiensis, B. dolosa, B. ambifaria, B. anthinia and B. pyrocinia*^18^. While MLST allowed us to type and establish their clonal groups and multilocus sequence analysis (MLSA) allowed us to infer phylogenetic relationship amongst the isolates and species of Bcc isolates. MLSA revealed that vast majority of the isolates belonged to *B. cenocepacia* and form one lineage, the remaining few belonged to *B. cepacia* and form a minor lineage. All three CF isolates belong to the *B. cenocepacia*. Interestingly, one CF isolate (1236) that also belongs to a novel ST forms out-group of dominant lineage suggesting that CF isolates can be more diverse or ancestral. Two serial isolates with identical STs from different tissues at the interval of one week from the CF patient suggests the chronic infection, which is a classic example of cepacia syndrome^23^. Hence, future studies will be aimed to look at more CF isolates for the extent of diversity and novel STs. Seven STs which comprises 21 isolates from our study are linked to 31CC, which is the largest clonal complex among all STs reported in the database. ST28 and ST32 strains are distributed globally to distinct locations and belong to clonal complex 31^18^. This suggests that isolates from our study are genetically closely related to these internationally spread clones. Analysis of recombination suggests that the population of Bcc is clonal in nature and mutations playing an important role rather than recombination in generating the genotyping diversity, however, few allelic loci exhibiting intergenic recombination. There is no evidence for the interspecies recombination but a larger collection of strains is required to investigate interspecies recombination events.

## Conclusion

Overall, MLST and MLSA analysis provided major insights into the diversity of Bcc population in India. There is a need to extend the study to larger collection from several more hospitals to encompass the whole of India for effective epidemiological and surveillance studies. There is also an immediate need to undertake such studies exclusively on CF patients. Further whole genome sequencing studies on this collection of Bcc is required to understand genomic diversity, variation by horizontal gene transfer, candidate virulence loci and its resistome.

## Material and Methods

### Ethical clearance

Ethical clearance was obtained from Institute Ethics committee at PGIMER, Chandigarh (Micro/2008/75, Dated 07.01.2008).

### Bacterial strains

We had screened clinical isolates collected from various specimens that included blood cultures, pus, respiratory specimens, sterile body fluids, intravenous catheters and cerebrospinal fluid (CSF), which were stocked over the last 7 years (2005–2012). Gram-negative, oxidase-positive, motile, NFGNB were selected and the identification of isolates as members of the Bcc was carried out by a triphasic analysis as, growth on the *B. cepacia* selective agar (BCSA), biochemical tests like oxidase, lysine decarboxylase, ornithine decarboxylase, arginine dihydrolase, triple sugar iron (TSI) and restriction fragment length polymorphism (RFLP) of *recA* gene^24^. Blood agar and MacConkey agar were used for routine isolation of Bcc. *Burkholderia cepacia* selective medium (BCSA) was used for the recovery of Bcc from respiratory specimens of CF patients that included sputum, induced sputum, bronchioalveolar lavage etc. which are diagnosed as per the guidelines of CF Foundation^25^. Bcc isolates were lyophilized and stored at 4 °C for further reference.

### Genomic DNA extraction, MLST locus amplification and sequencing

E-MLST was performed by sequencing of the 7 housekeeping genes: ATP synthase beta chain (*atpD*), glutamate synthase large subunit (*gltB*), DNA gyrase subunit B (*gyrB*), recombinase A (*recA*), GTP binding protein (*lepA*), acetoacetyl-CoA reductase (*phaC*), Tryptophan synthase subunit B (*trpB*) according to previously published method and available at www.pubMLST.org/bcc 11,12. The list of primer used in the MLST locus amplification is mentioned in Supplementary Table 4. Genomic DNA was isolated by using QIAamp DNA Mini Kit (Qiagen, Hilden, Germany) according to the manufacturer’s instructions for bacterial cells. Amplification of MLST locus was performed in 25-μl PCR reaction mixture volumes containing a final concentration of 2 mM MgCl_2_, 20 mM Tris-HCl, 50 mM KCl, 250 μM of each deoxynucleoside triphosphate, 0.4 μM of each primer, 1 M betaine, 10% dimethyl sulfoxide (Sigma-Aldrich) and 2U of Taq polymerase (Sigma-Aldrich). Amplification was performed with Applied Biosystems (Applied Biosystems, Thermo Scientific Company, Waltham, MA, USA) thermocycler system with conditions: initial denaturation for 2 min at 95 °C and 30 cycles were performed, each consisting of 30 s at 94 °C, 30 s at annealing temperature ranging from 55–60 °C (optimal annealing temperature for each locus is mentioned in Supplementary Table 4), and 60 s at 72 °C, followed by a final extension step of 5 min at 72 °C. The amplified product was run on the 1.2% agarose gel followed by purification using QIAquick Gel Extraction Kit (Qiagen, Hilden, Germany) following manufacturer’s protocol before being used in a sequencing reaction. Using sequencing primers, nucleotide sequences were determined at least once on each DNA strand with the BigDye Terminator ready reaction mix, version 3.1 (Applied Biosystems, Thermo Fisher Scientific Company, Waltham, MA.) under standard sequencing conditions according to the manufacturer’s protocol. Unincorporated dye terminators were removed by precipitation with 95% alcohol. The reaction products were separated and detected on an ABI PRISM genetic analyzer 3100 (Applied Biosystems, Thermo Fisher Scientific Company, Waltham, MA) using a standard sequencing module with a performance-optimized polymer and 5-cm array. The sequences from both strands of a given locus of the same isolate were aligned, trimmed to the desired length and edited using SeqMan II program from the Lasergene software package (DNASTAR, Inc., Madison, WI).

### Expanded Multi-Locus Sequence Typing (E-MLST)

Allele profiles, sequence types (ST) and clonal complexes were assigned by using the E-MLST database (www.pubMLST.org/bcc/). Alleles and ST that had not been previously described were submitted to the database and were assigned new allele numbers and STs.

### Population structure analysis

The STs generated in MLST were subjected to the eBURST analysis by eBURSTv3 in order to assign them into the Clonal complex/Burst groups (BG). The eBURSTv3 is based on a model of bacterial evolution whereby a single ancestor founder ST undergoes diversification to produce a subset of closely related STs and available at http://eburst.mlst.net/26. The relationship between the STs generated in this study with the existing STs in the global MLST database was assessed by using geoBURST^27^.

### Sequence diversity analysis

The number of polymorphic sites, nucleotide diversity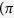), average GC content was calculated by using DnaSP Version 5.10^28^. For calculation of the average non-synonymous/synonymous substitution rate ratios (dN/dS), we first corrected the allelic locus of *gyrB, atpD* and *gltB* in order to get them in translating frame and dN/dS ratio was calculated by using MEGA version 6.06^29^.

### Multilocus sequence analysis

The concatenated nucleotide sequences of seven MLST allelic loci of reference strains of Bcc members (Supplementary Table 2) was retrieved from the PubMLST (www.pubMLST.org/bcc/). Sequence alignment of the concatenated seven housekeeping gene fragments (E-MLST) of all isolates and reference strains of Bcc members was carried out and Maximum Likelihood tree was constructed by using General Time Reversible model, Gamma distributed and Invariant sites (G + I) with 1000 bootstrap replications using MEGA version 6.06^29^.

### Split network and Recombination analysis

The split network of STs and individual loci was generated by using neighbor-net method^30^ using SplitTree4^31^. The pairwise homoplasy index (*phi)* test^32^ implemented in SplitTree4^31^ for recombination was performed, and the P value < 0.05 indicated recombination existed. LDhat programme^33^ implemented in Recombination Detection Program (RDP) v.4.66^34^ was used to calculate per site ρ/θ ratio based on the concatenated sequences of seven loci with 1,000,000 MCMC updates. The parameters ρ and θ represents the rate of recombination and mutation respectively. Linkage disequilibrium from allelic data was evaluated by calculating the standardised index of association (*I*_*A*_^*S*^) using LIAN v3.7^35^ (http://adenine.biz.fh-weihenstephan.de/lian_3.1/). The null hypothesis of complete linkage equilibrium (*I*_*A*_^*S*^ > 0; presence of linkage disequilibrium or clonality) was tested by using Monte Carlo methods with 10,000 iterations on allelic profile.

## Additional Information

**How to cite this article**: Gautam, V. *et al*. Multilocus sequence analysis reveals high genetic diversity in clinical isolates of *Burkholderia cepacia* complex from India. *Sci. Rep.* **6**, 35769; doi: 10.1038/srep35769 (2016).

## Supplementary Material

Supplementary Information

## Figures and Tables

**Table 1:** Nucleotide and allelic diversity of MLST loci.

Locus	Length (bp)	Average G + C content (%)	No. of alleles	No. of polymorphic sites	Average dN/dS ratio (%)	Nucleotide diversity (π)
*atpD*	443	62.4	7	19	0.241249	0.00587
*gltB*	400	67.5	7	13	0.025041	0.00522
*gyrB*	454	63.8	19	46	0.052232	0.02003
*lepA*	397	65.9	5	15	0.02288	0.00689
*phaC*	385	61.1	7	18	0.003964	0.00635
*recA*	393	68.3	6	42	0.023996	0.01755
*trpB*	301	69.3	7	20	0	0.01274

**Table 2:** Results of tests for the recombination.

Population(n)	*Phi* test	Recombination analysis					Linkage disequilibrium	
		theta/site	rho/site	rho/theta	LB 95%	UB 95%	I_A_^S^	P value
All (n = 21STs)	5.923E-4	1.609E-02	2.513E-03	0.1561	1.881E-03	3.379E-03	0.3828	<1.00E-04
L1 (n = 17STs)	0.2992	8.439E-03	1.087E-05	1.289E-03	1.065E-05	1.118E-05	0.3828	<1.00E-04
L2 (n = 4STs)	0.1748	8.828E-03	1.384E-03	0.1567	7.421E-04	2.041E-03	0.2733	9.8E-02
