# Supplementary material for: Multilocus sequence analysis reveals high genetic diversity in clinical isolates of Burkholderia cepacia complex from India
Source: Sci Rep. 2016 Oct 21;6:35769. doi: 10.1038/srep35769 (PMC5073313; doi:10.1038/srep35769)
Supplement: Supplementary Information [file srep35769-s1.pdf]

Supplementary information

Multilocus sequence analysis reveals high genetic diversity in clinical isolates of *Burkholderia cepacia* complex from India

Vikas Gautam[1*], Prashant P Patil[2], Sunil Kumar[1], Samriti Midha[2], Mandeep Kaur[1], Satinder Kaur[1], Meenu Singh[3], Swapna Mali[4], Jayanthi Shastri[4], Anita Arora [5], Pallab Ray[1], Prabhu B Patil[2*]

[1]Department of Medical Microbiology Post Graduate Institute of Medical Education and Research, Chandigarh, India.

[2]Bacterial Genomics and Evolution Laboratory, CSIR-Institute of Microbial Technology, Chandigarh, India.

[3]Department of Paediatrics, Post Graduate Institute of Medical Education and Research, Chandigarh, India.

[4]Department of Microbiology, Topiwala National Medical College & B. Y. L. Nair Charitable Hospital, Mumbai, India.

[5]Fortis Escorts Heart Institute, New Delhi, India.

*Corresponding author : VG (r_vg@yahoo.co.uk) or PBP (pbpatil@imtech.res.in)

Supplementary Table 1

Burkholderia cepacia complex (Bcc) strain collection used in this study, their isolation details along with allelic profiles and sequence type identified.

	Isolate ID	Source	Year	RFLP	Location	*atpD*	*gltB*	*gyrB*	*recA*	*lepA*	*phaC*	*trpB*	ST
1	29522	Blood	2011	G	PGIMER	131	11	487	14	11	6	79	628
2	33505	Blood	2011	G	PGIMER	131	11	487	14	11	6	79	628
3	22162	Blood	2007	G	PGIMER	131	11	487	14	11	6	79	628
4	9500	Blood	2007	G	PGIMER	131	11	487	14	11	6	79	628
5	1309	Respi	2011	G	PGIMER	131	11	487	14	11	6	79	628
6	1288	Respi	2011	G	PGIMER	131	11	487	14	11	6	79	628
7	4654	Respi	2010	G	PGIMER	131	11	487	14	11	6	79	628
8	25980	Blood	2010	G	PGIMER	15	11	487	14	11	6	79	839
9	30379	Blood	2011	G	PGIMER	15	11	487	14	11	6	79	839
10	30380	Blood	2011	G	PGIMER	15	11	487	14	11	6	79	839
11	30879	Blood	2011	G	PGIMER	15	11	487	14	11	6	79	839
12	31369	Blood	2011	G	PGIMER	15	11	487	14	11	6	79	839
13	32670	Blood	2011	W	PGIMER	131	394	585	14	11	6	79	827
14	15 ED	Blood	2008	G	FEHI	131	11	187	143	11	6	79	843
15	2 ED	Blood	2008	G	FEHI	15	11	187	143	11	6	79	232
16	8 ED	Blood	2008	G	FEHI	15	11	187	143	11	6	79	232
17	20 ED	Blood	2008	G	FEHI	15	11	187	143	11	6	79	232
18	31 ED	Blood	2008	G	FEHI	15	11	187	143	11	6	79	232
19	44 ED	Blood	2008	G	FEHI	15	11	187	143	11	6	79	232
20	23186	Blood	2007	G	PGIMER	131	11	174	14	11	6	79	217
21	64329	Blood	2012	G	GHH	131	11	174	14	11	6	79	217
22	7055	Blood	2010	G	PGIMER	15	11	582	14	11	6	147	826
23	7716	Respi	2010	G	PGIMER	15	11	582	14	11	6	147	826
24	32127	Blood	2012	G	PGIMER	131	11	230	14	11	6	358	709
25	32358	Blood	2012	W	PGIMER	131	11	230	14	11	6	358	709
26	31674	Blood	2012	G	PGIMER	131	11	230	14	11	6	358	709

27	4671	Fluid	2010	G	PGIMER	15	11	581	14	11	141	79	824
28	BMU	Urine	2012	G	TNMC & BYL	15	11	581	14	11	141	79	824
29	18963	Blood	2010	G	PGIMER	15	394	184	14	11	6	79	832
30	18965	Blood	2010	G	PGIMER	15	394	184	14	11	6	79	832
31	2722	Pus	2009	G	PGIMER	340	11	442	14	11	6	79	829
32	22565	Blood	2005	G	PGIMER	16	11	179	14	11	120	79	208
33	49365	Blood	2012	W	GHH	15	11	481	14	11	6	147	621
34	551	ETA	2012	G	PGIMER	16	11	271	143	11	6	147	807
35	1125	Blood	2010	G	PGIMER	16	11	271	143	11	6	147	807
36	1626	Blood	2011	G	PGIMER	16	11	271	143	11	6	147	807
37	2321	ETA	2012	G	PGIMER	16	11	271	143	11	6	147	807
38	29416	Blood	2009	G	PGIMER	16	11	271	143	11	6	147	807
39	31759	Blood	2011	G	PGIMER	16	11	271	143	11	6	147	807
40	32447	Blood	2012	G	PGIMER	16	11	271	143	11	6	147	807
41	35238	Blood	2012	G	PGIMER	16	11	271	143	11	6	147	807
42	1168	Respi	2011	G	PGIMER	15	11	580	14	11	6	147	822
43	31704	Blood	2011	G	PGIMER	15	11	487	14	11	6	79	839
44	7216	Blood	2010	Neg	PGIMER	15	11	582	14	11	6	147	826
45	1236	Respi	2010	I	PGIMER	16	16	588	19	68	278	13	828
46	7142	Blood	2006	AN	PGIMER	15	394	184	14	11	6	79	832
47	4613	Blood	2010	E	PGIMER	339	393	587	37	1	93	48	825
48	31178	Blood	2011	W	PGIMER	91	93	96	103	42	1	21	9
49	5310	Blood	2010	E	PGIMER	339	393	587	37	1	93	48	825
50	5312	Blood	2010	E	PGIMER	339	393	587	37	1	93	48	825
51	8947	Blood	2007	E	PGIMER	66	109	49	3	3	40	53	6
52	66992	Blood	2012	W	GHH	1	386	344	3	3	1	53	840
53	BC-3	Blood	2012	G	TNMC & BYL	15	11	593	11	6	14	79	841
54	BC-19	Blood	2012	Neg	TNMC & BYL	15	11	581	14	11	141	79	824
55	BC-21	Blood	2012	G	TNMC & BYL	15	11	581	14	11	141	79	824
56	BC-40	Blood	2012	Neg	TNMC & BYL	15	11	581	14	11	141	79	824
57	BC-41	Blood	2012	Neg	TNMC & BYL	15	11	581	14	11	141	79	824

W- Faint bands of *recA* PCR were observed

Neg: Negative

Respi: Respiratory specimen, ETA: Endotracheal aspirate

PGIMER: Post-graduate Institute of Medical Education and Research, Chandigarh.

FEHI: Fortis Escorts Heart Institute, New Delhi.

TNMC & BYL: TNMC & BYL Nair, B. Y. L. Nair Charitable Hospital, Mumbai.

GHH: Global Hospitals & Health City, Chennai.

Supplementary Table: 2

e-BURST analysis result of 21 STs from 57 Bcc isolates under study.

Group 1: No. Isolates = 15 | No. STs = 3 | Predicted Founder = 628									
					Average		ST Bootstrap		
ST	FREQ	SLV	DLV	TLV	SAT	Distance		Group	Subgrp
628	7	2	0	0	0	1.0	30%	0%	
839	6	1	1	0	0	1.5	0%	0%	
217	2	1	1	0	0	1.5	0%	0%	
Group 2: No. Isolates = 5 | No. STs = 3 | Predicted Founder = 826									
					Average		ST Bootstrap		
ST	FREQ	SLV	DLV	TLV	SAT	Distance		Group	Subgrp
826	3	2	0	0	0	1.0	8%	0%	
621	1	2	0	0	0	1.0	5%	0%	
822	1	2	0	0	0	1.0	18%	0%	
Group 3: No. Isolates = 6 | No. STs = 2 | Predicted Founder = None									
ST	FREQ	SLV	DLV	TLV	SAT	Distance			
232	5	1	0	0	0	1.0			
843	1	1	0	0	0	1.0			
Singletons: size 13									
807									
832									
709									
841									
840									
9									
829									
208									
828									
827									
6									
825									
824									

Supplementary Table 3

List of reference strains of Bcc members and their sequence types used in MLSA analysis.

Sr. No	Species name	Sequence Type
1)	*B. cepacia* ATCC 25416^{T}	ST-10
2)	B. cenocepacia J2315^{T}	ST-28
3)	*B. ambifaria* AMMD^{T}	ST-77
4)	*B. multivorans* LMG 13010^{T}	ST-397
5)	*B. dolosa* AU0645 ^{T}	ST-72
6)	*B. vietnamiensis* LMG 10929^{T}	ST-65
7)	*B. pyrrocinia* ATCC 15958^{T}	ST-41
8)	*B. ubonensis* LMG 20358^{T}	ST-299
9)	*B. stabilis* LMG 14294^{T}	ST-50
10)	*B. anthina* LMG 20980^{T}	ST-86
11)	*B. contaminans* LMG 20980^{T}	ST-102
12)	*B. lata* ATCC 17760^{T}	ST-101
13)	*B. diffusa* LMG 24065^{T}	ST-164
14)	*B. latens* LMG 24064^{T}	ST-104
15)	*B. arboris* ES0263A^{T}	ST-492
16)	*B. Seminalis* AU0475^{T}	ST-473
17)	*B. metallica* AU0553^{T}	ST-511
18)	*B. pseudomultivorance* AU3207^{T}	ST-511
19)	*B. stagnalis* LMG 285156^{T}	ST-787
20)	*B. territorii* LMG 28158^{T}	ST-791

Supplementary Table 4

Primer sequences with annealing temperatures used for the amplification and sequencing of seven MLST loci of *Burkholderia cepacia complex* isolates.

**MLST Locus**	**Amplicon size (bp)**	**Primer sequence (5'- 3')**	**Annealing temp (°C)**
atpD	756	ATGAGTACTRCTGCTTTGGTAGAAGG<br>CGTGAAACGGTAGATGTTGTCG	55
gltB	652	CTGCATCATGATGCGCAAGTG<br>CTTGCCGCGGAARTCGTTGG	60
gyrB	738	ACCGGTCTGCAYCACCTCGT<br>YTCGTTGWARCTGTCGTTCCACTGC	60
lepA	975	CTSATCATCGAYTCSTGGTTCG<br>CGRTATTCCTTGAACTCGTARTCC	55
phaC	525	GCACSAGYATYTGCCAGCG<br>CCATSTCSGTRCCRATGTAGCC	60
recA	704	AGGACGATTCATGGAAGAWAGC<br>GACGCACYGAYGMRTAGAACTT	60
trpB	787	CGCGYTTCGGVATGGARTG<br>ACSGTRTGCATGTCCTTGTCG	60
